# Knowledge Is Power: Prior Knowledge Aids Memory for Both Congruent and Incongruent Events, but in Different Ways

**DOI:** 10.1037/xge0000498

**Published:** 2018-11-05

**Authors:** Andrea Greve, Elisa Cooper, Roni Tibon, Richard N. Henson

**Affiliations:** 1 MRC Cognition & Brain Sciences Unit, University of Cambridge

**Keywords:** prediction error, schema, associative memory, encoding, one-shot learning

## Abstract

Events that conform to our expectations, that is, are congruent with our world knowledge or schemas, are better remembered than unrelated events. Yet events that conflict with schemas can also be remembered better. We examined this apparent paradox in 4 experiments, in which schemas were established by training ordinal relationships between randomly paired objects, whereas event memory was tested for the number of objects on each trial. Better memory was found for both congruent and incongruent trials, relative to unrelated trials, producing memory performance that was a “U-shaped” function of congruency. The congruency advantage but not incongruency advantage was mediated by postencoding processes, whereas the incongruency advantage, but not congruency advantage, emerged even if the information probed by the memory test was irrelevant to the schema. Schemas therefore augment event memory in multiple ways, depending on the match between novel and existing information.

More than 80 years ago, Bartlett (1932) observed that memory for new information is better if that information fits with prior knowledge, or what he called a “schema” (see also Anderson, 1981; Rumelhart, 1980; Tse et al., 2007; van Kesteren, Ruiter, Fernández, & Henson, 2012). In contrast, other studies have reported the opposite finding: superior memory for information that is unexpected, for example by virtue of being incongruent with a schema (e.g., Greve, Cooper, Kaula, Anderson, & Henson, 2017; Mäntylä & Bäckman, 1992; Tulving & Kroll, 1995; Von Restorff, 1933). However, these seemingly paradoxical findings are typically observed under quite different conditions, and it is unclear whether they are mediated by different psychological processes. The present aim was to bring together these two areas of research by demonstrating the congruency advantage and incongruency advantage simultaneously within the same paradigm and identify factors that dissociate these “two extremes” of the congruency continuum.

There are numerous demonstrations in the literature of the ability of prior knowledge to facilitate memory for events congruent with that knowledge, that is, that conform to our expectations. This congruency effect (Alba & Hasher, 1983) has been reported for a wide range of stimuli and modalities, such as item-color pairs (Cycowicz, Nessler, Horton, & Friedman, 2008), scene-item pairs (Liu, Grady, & Moscovitch, 2018; van Kesteren et al., 2013), item-location pairs (Atienza, Crespo-Garcia, & Cantero, 2011; van Buuren et al., 2014), and relational information across items (Ostreicher, Moses, Rosenbaum, & Ryan, 2010), but also word lists (Bein et al., 2015; Craik & Tulving, 1975; Packard et al., 2017), stories (Bransford & Johnson, 1972; Mandler & Johnson, 1977), films (van Kesteren, Fernández, Norris, & Hermans, 2010), and other multisensory stimuli (Heikkilä, Alho, Hyvönen, & Tiippana, 2015; Heikkilä, Alho, & Tiippana, 2017; Moran et al., 2013). For instance, congruent color-item pairings, such as the word *tomato* presented on a red background, are more likely to be remembered than incongruent pairings. This event memory (Rubin & Umanath, 2015) advantage has been attributed to easier integration of information that matches representations in semantic memory, enabling the formation of richer, more elaborated representations that are more easily accessible during retrieval (Craik & Tulving, 1975). At the same time, information that is not relevant to the current schema is often remembered less well (Sweegers, Coleman, van Poppel, Cox, & Talamini, 2015; Sweegers, Takashima, Fernández, & Talamini, 2014), that is, a schema can both help and hinder memory for new events.

An equally long-standing but somewhat separate line of research, however, has firmly established the apparent opposite finding, by which events incongruent with our expectations are remembered better (e.g., Greve et al., 2017; Hunt & Worthen, 2006; Tulving & Kroll, 1995; Von Restorff, 1933). A cardinal example of this is the “isolation effect” described by Von Restorff (1933), whereby an item that is distinct from other items in a list is better recalled. For example, when a list of words is presented in the same format except for one (e.g., the word tomato in a list “APPLE, PEAR, ORANGE, tomato, BANANA”, etc.), the probability of later recalling that “tomato” was in the list is normally higher than if it had appeared in uppercase. Schmidt (1991) called this an example of “primary distinctiveness,” where an item differs perceptually or conceptually from other items close in time or space. He distinguished this from “secondary distinctiveness,” where items appear unusual according to general knowledge (McDaniel & Einstein, 1986). For example, the word tomato in the sentence “her blue handbag contained a tomato” is unexpected because of our prior knowledge about what handbags contain, which comes from more than the immediately surrounding words. In other words, we have a schema that handbags do not normally contain fruit. That participants presented with this sentence are likely to remember the tomato would seem, at a first glance, to contradict the above claim that only information that conforms to a schema is better remembered. Note, however, that the tomato is not simply irrelevant to the schema about what handbags contain—it is actually incongruent with that schema. This is in contrast to the sentence “her blue bag contained a tomato,” where the more generic concept of a bag (rather than a handbag) renders the tomato neither particularly congruent nor incongruent. It is this need to consider more than two levels of congruency that we propose reconciles the apparent paradox, because it enables one to demonstrate a nonlinear (U-shaped) relation between memory and congruency, in which highly congruent and highly incongruent events are both remembered better than less (in)congruent events. Thus, whether one finds better memory for congruent or incongruent events when comparing just two levels of congruency depends where those levels fall on the U-shaped function. This was the basis of the experimental paradigm we developed here.

Before introducing our paradigm, it is worth considering other factors that may be important for understanding the mnemonic advantages afforded to congruent and incongruent information. One question central to memory research on both schema and the isolation effect is whether the mnemonic advantage arises at encoding and/or retrieval. Research on schema for example has shown that providing a schema after initial encoding of target information can still help recall of that information, suggesting that schema can help organize retrieval (or rehearsal), as well as initial encoding (Anderson, Pichert, & Shirey, 1983). Furthermore, in the example of a sentence “her blue handbag contained a wallet,” later memory for the object “wallet” can be facilitated by using prior knowledge at retrieval to generate likely objects found in a handbag, until one is recognized from the prior sentence—a so-called “generate-and-recognize” strategy (Watkins & Gardiner, 1979).

Early accounts of the isolation effect, on the other hand, argued that it is the surprise that participants experience due to a physical or conceptual change that attracts additional attention and thereby facilitates encoding (R. T. Green, 1956; Jenkins & Postman, 1948; Rundus, 1971). However, these accounts are difficult to reconcile with examples that an isolation effect, based on primary distinctiveness, can occur even for the first few items in a list (Dunlosky, Hunt, & Clark, 2000). This finding (in fact already reported in the seminal paper by Von Restorff, 1933; see also Hunt, 1995) is difficult to explain in terms of encoding processes because expectations must be formed by a number of prior items before they can be violated by an isolate. This led to alternative accounts that emphasize the role of distinctiveness at retrieval (Dunlosky et al., 2000; Hunt, 1995; Kelley & Nairne, 2001). Bruce and Gaines (1976), for instance, show that words that are distinct by being physically isolated are clustered together during recall, suggesting distinct items might be stored and retrieved together as a special “unusual item” category. Another view proposes that distinctiveness is directly linked to the retrieval context, which provides an advantage in the processes involved in retrieving items from that specific context (McDaniel, Einstein, DeLosh, May, & Brady, 1995). Nonetheless, more recent work (Schmidt & Schmidt, 2017) has demonstrated that unexpected events benefit from processes operating at both encoding and retrieval, depending on the context and task demands.

Another consideration is whether the mnemonic advantage afforded to congruent and incongruent information arises from the same or different mechanisms. Here, recent evidence from neuroscience is relevant, in suggesting that different brain systems support memory at these two extremes of the congruency continuum. Many years of research have implicated the medial temporal lobes (MTL), and the hippocampus in particular, in event memory, and some have proposed that the hippocampus is especially sensitive to novelty or prediction errors (Nyberg, McIntosh, & Tulving, 1998; Strange, Duggins, Penny, Dolan, & Friston, 2005; Kumaran & Maguire, 2006, 2009). This suggests that the MTL might be important for encoding incongruent information. Standard consolidation theory then states that, after initial encoding in the hippocampus, information is subsequently transferred into the cortex for longer-term storage (e.g., during sleep; Buzsáki, 1998; Davis & Gaskell, 2009; Ji & Wilson, 2007; Marshall & Born, 2007; Squire & Alvarez, 1995). However, research in rodents has shown that information can be consolidated into cortex much more rapidly when it is congruent with a schema (Tse et al., 2007). The medial prefrontal cortex (mPFC) seems particularly important for this rapid cortical learning of congruent information (Tse et al., 2011), which is supported by neuroimaging findings in humans (Bein et al., 2015; Brod, Lindenberger, Werkle-Bergner, & Shing, 2015; Preston & Eichenbaum, 2013; van Kesteren et al., 2010, 2013).

These findings provided the foundation for a theoretical framework called *schema-linked interactions between medial prefrontal and medial temporal lobes* (SLIMM), which integrates schema theory with recent neuroscientific data (van Kesteren et al., 2012). SLIMM defines a schema as an active set of mutually reinforcing neocortical representations, which affect online processing. A new event that is incongruent with that schema causes a prediction error that triggers an MTL system (containing the hippocampus) to encode that event, including contextual details that are not directly relevant to the current schema (van Kesteren et al., 2012). This proposal is based on neuroscientific evidence that links the MTL to the acquisition and integration of event information (Eichenbaum, Yonelinas, & Ranganath, 2007). For example, the detail that the handbag was blue should be better remembered in the incongruent example “her blue handbag contained a tomato” than the congruent example “her blue handbag contained a wallet.” Encoding all details in a surprising event has adaptive value in order to help identify what might have caused the prediction error, and hence update schemas to allow more accurate predictions in future (e.g., Are blue handbags a special type of handbag?).

By contrast, events that are congruent with the current schema are hypothesized to trigger a system based in the mPFC that then enables rapid learning in the neocortex (independent of the hippocampus), though only for those details of the event that are congruent with the schema. More specifically, mPFC is hypothesized to detect the degree of “resonance” between the perceptual input and the cortical representations already active (the schema). When the resonance is high, mPFC is assumed to suppress the MTL system, so that perceptual input (event detail) that is unrelated to the schema is not encoded. This leaves the perceptual input that is congruent with the schema, which is then rapidly learned directly within the cortex. The latter is consistent with claims, contrary to standard consolidation theory, that new information can be learned in patients with hippocampal damage, provided it is consistent with a schema (Gilboa & Marlatte, 2017).

Inspired by this neuroscientific SLIMM framework, we set out to test its behavioral predictions. The first prediction is a U-shaped function of event memory against congruency, with best memory at the two extremes of highly congruent and highly incongruent. The second and third predictions concern ways in which these two extremes are functionally dissociable. One way that they should dissociate concerns whether they arise during encoding or retrieval. SLIMM predicts that the incongruency advantage arises at encoding, when prediction errors drive the MTL to store a complete representation of the surprising event, whereas the congruency advantage can also occur after encoding. The latter can arise for two reasons. First, a schema might be used to generate congruent information at test, as in the “generate-and-recognize” strategy described above. However, we deliberately designed our paradigm to rule out this somewhat trivial mechanism. Second, SLIMM hypothesizes that congruent information is more likely to be reactivated after initial encoding than is incongruent information, which would strengthen memories before they are tested. We therefore examined memory for events that occurred before a schema was established and predicted that they would be better remembered when congruent with the subsequent schema, but not when incongruent. The second way that the two extremes of the U-shape should dissociate (and the third prediction of SLIMM) concerns memory for incidental details of the encoded event. This should be improved for incongruent events (because prediction errors drive the MTL system to store the full event), but not for congruent events (because mPFC suppresses the MTL system such that details irrelevant to the schema are ignored).

We created a paradigm that manipulates the strength of a schema and whether events match that schema, resulting in three conditions: incongruent, unrelated and congruent. An analogy would be the earlier examples of “her blue handbag contained a tomato” (incongruent), “her blue bag contained a tomato” (unrelated) and “her blue handbag contained a wallet” (congruent). However, rather than using prior knowledge, we trained people through feedback to learn schemas during the experiment. Each schema was a simple rule that related two types of objects, which is arguably the simplest form of a schema, in the sense of an abstract structure that can apply to different events. By training schemas experimentally, rather than relying on pre-experimental knowledge, we could examine memory for events both before and after a schema had been learned. The events themselves were individual trials that varied in the number of exemplars of each type of object (each trial had a unique combination of exemplar numbers). Importantly, when testing recognition memory for those trials, the number of exemplars could not be inferred or guessed based on knowledge of the rule, that is, any memory advantage for trials that were congruent with a schema could not occur solely by applying the schema at retrieval. Furthermore, by making the number of exemplars relevant or irrelevant to the schema, we could test memory for information that is incidental to the schema. We ran four experiments that varied these different aspects of the paradigm.

## Overview of Experiments

**Figure 1 fig1:**
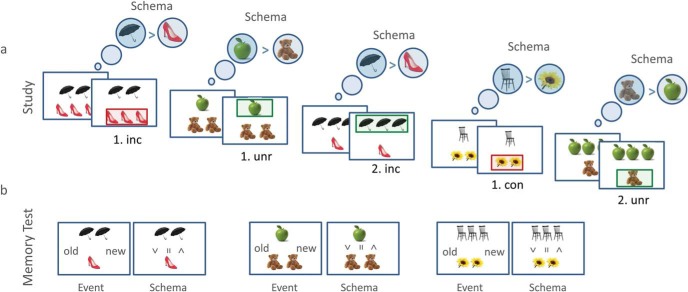
Illustration of the general procedure. On each trial in the study phase (a), participants saw exemplars of two types of object, one above the other. The number of exemplars of each object varied across trials, with specific combinations of numbers (e.g., two umbrellas and three shoes) only occurring once. Participants had to choose the set of objects with higher value, having been told that one of the objects had twice the value of the other (e.g., umbrellas being twice as valuable as shoes), but initially not knowing which. They learned this schema through trial-and-error, with feedback after their choice provided by a green (correct) or red (incorrect) rectangle. For example, if they chose three shoes over two umbrellas and got negative feedback, then they could infer that the umbrella was the more valuable object. Occasionally the schema reversed. The number and timing of these schema reversals determined the three conditions—incongruent (inc), unrelated (unr), and congruent (con)—see Figure 2. The number of exemplars of each object was relevant to the task in Experiments 1–3, which was to determine the higher total value (object value times number of exemplars), but irrelevant in Experiment 4, where the higher value object was independent of the number of exemplars. The three conditions were intermixed (and no object appeared in more than one condition). During the subsequent test phase (b), “old” displays from some study trials were shown, intermixed with “new” displays that contained new combinations of the same objects. Two decisions were required for each display: (a) whether the display had been seen at study (i.e., the precise numbers of each object) and (b) which set of objects was more valuable (as during study). The first decision provided an index of event memory whereas the second decision, for new displays at least, measured how well the schema had been learned.

**Figure 2 fig2:**
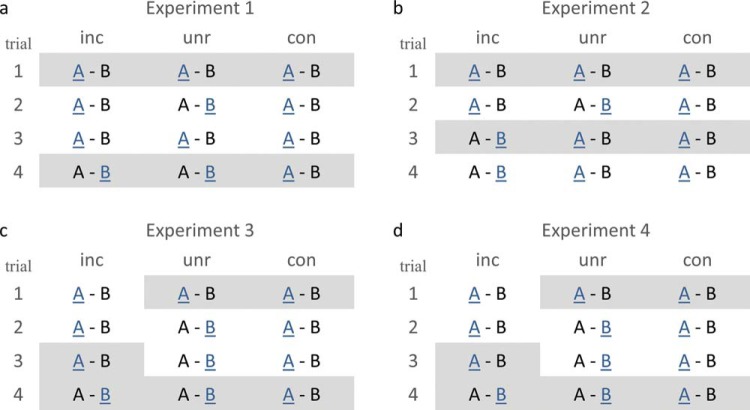
Illustration of the design of each experiment. Objects are abbreviated as A and B and for simplicity their numbers are not shown. The more valuable object according to the current schema is shown in blue and underlined. Each repetition of a given pair of objects (Trials 1–4) conformed to the same schema for the congruent (con) condition, but reversed on the third trial (Experiment 2) or last trial (Experiments 1, 3, and 4) in the incongruent (inc) condition. The schema changed more frequently in the unrelated (unr) condition, depending on the experiment (see text). Trials for which memory was later tested are shown with a gray background.

The basic paradigm for all four experiments is shown in Figure 1 and contained a study phase and test phase. At study (Figure 1a), participants learned via feedback which of two types of objects had a higher value (the rule). In congruent conditions, the rule remained constant across trials; in incongruent conditions, the rule reversed after the penultimate trial, that is, before the final critical trial that was later tested; in unrelated conditions, rules reversed after the first trial (and perhaps further trials, depending on the experiment; Figure 2). Thus in congruent and incongruent conditions, a schema could be established, which was either violated (incongruent condition) or not (congruent condition) on the critical trial (fourth trial in Experiment 1, 3 and 4; third trial in Experiment 2); whereas in unrelated conditions, only a weak schema, if any, could be learned. All study events were trial-unique, allowing subsequent assessment of event memory.[Fig-anchor fig1][Fig-anchor fig2]

At test, participants were shown “old” trials that were previously encountered during study, or “new” trials that involved a new combination of studied objects (Figure 1b). Two decisions were required for each test trial: (a) whether or not the display had been seen at study (i.e., the precise numbers of each object paired together in a trial) and (b) which set of objects was more valuable (the same task as during study). The primary outcome was the first decision, which assessed event memory (i.e., memory for a unique trial). The second decision provided, for new displays at least, a confirmation of how well schemas had been learned. Importantly, knowing the schema that applied to the objects in an test trial did not help to determine whether a specific combination of objects was previously studied.

Two kinds of “old” trials were tested in each condition. The first type of “old” trial tested memory for the last studied trial of each condition (the fourth trial in Experiment 1, 2, and 4; or third trial in Experiment 3). By the time of the last trial at study, expectations have been formed on the basis of the preceding trials, at least in the congruent and incongruent conditions. Such predictions are predicted to enhance memory for this trial either when violated (in the incongruent condition) or not (in the congruent condition). Memory for this trial should therefore reveal the predicted U-shaped function, with better memory for both the congruent and incongruent condition, compared to the unrelated condition.

The second type of “old” trial was the first encounter of a given pair of objects, that is, first study trial of each condition (with exception of incongruent trials in Experiment 3 and 4; see later). This type of test trial probed whether any memory advantage for congruent or incongruent trials arose at encoding or postencoding. Because the rule cannot have been learned prior to first occurrence of a pair of objects, any difference between conditions in subsequent memory for the first study trial should be driven by postencoding processes. According to the SLIMM framework, congruency effects can arise during postencoding reactivation, such that memory for the first trial should be better in the congruent than unrelated or incongruent conditions.

## Experiment 1

### Method

#### Participants

All experiments reported here recruited a unique set of Cambridge community members from the volunteer panel of the MRC Cognition and Brain Science Unit, all of whom had reported normal or corrected-to-normal visual acuity, provided informed consent and received monetary compensation for participation, as approved by a local ethics committee (Cambridge Psychological Research Ethics Committee reference 2005.08). Each volunteer only participated in one of the present experiments. The congruency effect has been previously investigated by Brod et al. (2015), who reported an effect size of *d* = 0.89 when testing 21 subjects. Similarly, Greve et al. (2017) tested 20 subjects and reported an incongruency effect of the size *d* = 0.57. On the basis of those reports and in the interest of assessing a fully counterbalanced design, we tested 24 participants (15 females; mean age 24 years, *SD* = 3.83 years), which provided 99% power to detect a congruency effect and 86% power to detect an incongruency effect using a one-tailed test.

#### Materials

Stimuli were 120 color images of distinct, everyday objects (https://osf.io/x692m/). They were divided into six sets of 20. Two sets were randomly assigned to each of the three conditions, counterbalanced across participants. Within each condition, trials were created by randomly selecting one object from each set and randomly assigning it a value of 1 and 2. One, two, or three exemplars of each object were selected for each of four trials, with the constraints that the combination of numbers did not repeat across trials and that the difference in number of exemplars of each object was no more than one (because larger differences, e.g., three umbrellas and one shoe, would not enable induction of the schema as umbrellas would always have greater collective values regardless). The initial assignment of objects to top or bottom of the screen was random but was kept constant across repeated trials.

#### Procedure

The general procedure for all experiments is illustrated in Figure 1, and the specific design for each Experiment is shown in Figure 2a. In Experiment 1 participants were presented with 20 blocks, each consisting of two phases: study and test. Participants had to learn a rule (schema) about the relative value of two objects presented in each study trial, and their memory for individual trials (events), together with their memory for the schema, was assessed in the test phase immediately following each study phase. Each phase consisted of trials from three conditions: congruent, where the rule was constant; unrelated, where the rule changed frequently making it difficult to learn; and incongruent, where the rule only changed on the last trial. Prior to the start of the experiment, participants completed a practice session to familiarize themselves with the procedure.

#### Study phase

Each study phase presented one unique object pair for each of the three conditions. Object pairs were seen four times (each trial with unique numbers of exemplars), so that a total of 12 trials were shown, randomly intermixed. Participants had to indicate which set of objects had greater, or if appropriate equal, collective value. Participants were told one object in each pair had twice the value of the other, but not which, and that the upper/lower position on the screen was irrelevant. For instance, for a display of two umbrellas on top and three shoes on the bottom, with umbrella assigned the value of 2 and shoe assigned the value of 1, the umbrellas are of higher combined value and hence the top display should be selected (top > bottom; Figure 1a). Both the assigned value and number of items presented are relevant for performing the task accurately. Value assignment remained the same across all trials in the congruent condition, though it reversed for that fourth and last trial in the incongruent condition and reversed for every individual trial in the unrelated condition (Figure 2a). Participants made their response via one of three buttons for top, bottom or equal. Some trials did not provide sufficient information to test the rule (e.g., one umbrella and three shoes), but such ambiguous trials never occurred on the critical trials (i.e., first and last trials in Experiment 1), to ensure that correct responses reflected the accurate object-value assignments. The stimuli remained on the screen until a response was given, followed by a feedback screen for 1000ms. Feedback was conveyed by a box around the set of objects with greater collective value, which was either green if that set had been chosen correctly, or red if the other set had been chosen incorrectly. A symbol (>, <, =) was also presented in the midline between the objects to help learn the schema. The next trial commenced following a blank screen of 250 ms.

#### Test phase

For the test phase, object pairs were again shown with one object above the other, as in the study phase. In each block, six of the test trials (“old” trials) were identical to those in the study phase (from trial numbers shown in Figure 2); three additional “new” trials contained the same object pairs as old trials, but in a combination of numbers different from any study trial. Thus, there were two old and one new trial per condition. Old and new trials were randomly intermixed. Two decisions were made for each trial: the first testing event memory and the second testing schema memory. The first decision about event memory was prompted by the question “old/new?,” which stayed on the screen, together with the object-pair, until participants responded with one of six response buttons for: guess old, think old, sure old, guess new, think new, sure new. Note that the objects themselves were always old, but the specific number of objects may or may not have been previously studied. Once a response was made, the second decision about schema memory was prompted by the question “wins?.” Participants decided the relative collective value of the two sets of objects (like at study), using the same six response options: guess top, think top, sure top; guess equal, think equal, sure equal; guess bottom, think bottom, sure bottom.

#### Study performance

If schema were learned in the study phase, we predicted (a) greater prediction accuracy for last than first trial of the congruent condition (con_4 > con_1); (b) worse prediction accuracy for the last than first trial in the incongruent condition (inc_4 < inc_1), due to accuracy being below chance in the final trial following the unexpected schema change; and (c) worse prediction accuracy for the last than first trial in the unrelated condition (unr_4 < unr_1), due to below chance performance for the final trial, assuming that participants adjusted their responses to the preceding trial (Figure 2a). Given that participants were presented with three response options (top, equal, bottom), random key presses would produce accuracy of 1/3. However, the instruction that one object had twice the value of the other allowed one response option to be excluded on each trial, because (a) in trials with two exemplars of one object and one exemplar of the other, the latter could never have higher collective value; (b) in trials with three exemplars of one object and two exemplars of the other, collective values could never be equal; and (c) in trials with the same number of objects, the collective values could never be equal. Thus, even without knowledge of the schema (i.e., which of the objects was more valuable), informed guessing would result in an accuracy of 1/2. Thus, for first trials for example, we expected performance to lie somewhere between .33 and .50.

#### Test performance: Event memory

The main predictions concerned the final trials of each condition, where we expected better event memory for incongruent than unrelated trials, and for congruent than unrelated trials, that is, the U-shaped pattern inc_4 > unr_4 < con_4. If schemas also act after encoding, for example during encoding of subsequent congruent trials, we also predicted better memory for the first congruent trial than first unrelated trial, that is, unr_1 < con_1.

To control for different biases across conditions to call trials “old” or “new” (e.g., if participants showed a tendency to call trials from the congruent condition “old,” even when they were not), memory performance was calculated by subtracting false alarm rates from hit rates. To reduce the impact of guesses, we report only high confidence responses here, though analyses of all responses revealed a similar pattern (see Supplemental Table S1 in the online supplemental material). Thus, the hit rate was the proportion of old trials correctly called “sure old,” and the false alarm rate was the proportion of new trials incorrectly called “sure old.”

#### Test performance: Schema memory

Schema memory was scored the same way as during study (see above), where correct performance was defined as the most consistent schema during the study phase (i.e., that determined the first three trials in the incongruent condition, rather than just the last trial). For conditions where each schema applied to an equal number of trials (two), correct performance was defined by the schema that applied to the last (fourth) trial. Note that the focus was on schema knowledge for the new trials, where responses could not be based on event memory.

Given our a priori directional predictions (as specified above), *p* values are one-tailed with an alpha level of *p* = .050 (though the vast majority survived two-tailed correction), unless stated otherwise. Point estimates of effect sizes and confidence intervals are presented by reporting the mean differences (MD) between conditions and their 95% confidence interval (CI), followed by Cohen’s d.

### Results

#### Schema learning and memory

Accuracy during study (see Table 1) significantly increased from the first to last congruent trial (con_4>con_1: *t*(23) = 14.42, *p* < .001, MD = .45, CI [.40, .50], *d* = 2.94), becoming significantly above chance (con_4 > .5: *t*(23) = 10.69, *p* < .001, MD = .37, CI [.31, .42], *d* = 2.18), but significantly decreased from first to last incongruent trial (inc_4<inc_1: *t*(23) = −7.80, *p* < .001, MD = −.33, CI [−.40, −.26], *d* = −1.60), falling below chance on the last trial when the schema was reversed, as expected (inc_4 < .5: *t*(23) = −14.14, *p* < .001, MD = −.37, CI [−.42, −.33], *d* = −2.89). Performance was significantly worse in the last than first unrelated trials (unr_4 < unr_1: *t*(23) = −1.98, *p* = .03, MD = −.09, CI [−.18, −.01], *d* = −0.40) and was below chance (unr_4 < .5: *t*(23) = −3.10, *p* = .003, MD = −.13, CI [−.20, −.06], *d* = −0.63), as predicted if participants were basing their decision on the previous trial in this condition. Indeed, performance for unr_4 trials did not differ from random key presses (unr_4 = .33: *t*(23) = 0.90, *p* = .19, MD = .04, CI [−.03, .11], *d* = 0.18) which could mean that participants “gave up” learning a schema in the unrelated condition due to the frequent rule reversals.[Table-anchor tbl1]

Schema memory for new trials at test (last row of Table 1) was significantly better in the congruent than incongruent condition, *t*(23) = 4.83, *p* < .001, MD = .12, CI [.08, .16], *d* = 0.99, and significantly better in the incongruent than unrelated condition, *t*(23) = 6.97, *p* < .001, MD = .21, CI [.16, .26], *d* = 1.42, as expected, with the latter not being significantly different from chance, *t*(23) = 0.63, *p* = .54, two-tailed, MD = .02, CI [−.05, .09], *d* = 0.13. These results confirm that participants learned schemas in the congruent and incongruent conditions, but not the unrelated condition.

#### Event memory: Last critical trial (4th trial)

The hit and false alarm rates in the test of event memory are shown in Table 2. To adjust for possible biases (e.g., toward calling trials in the congruent condition “old”), memory accuracy was defined as the difference between hit and false alarm rates (where zero means no memory).[Table-anchor tbl2]

**Figure 3 fig3:**
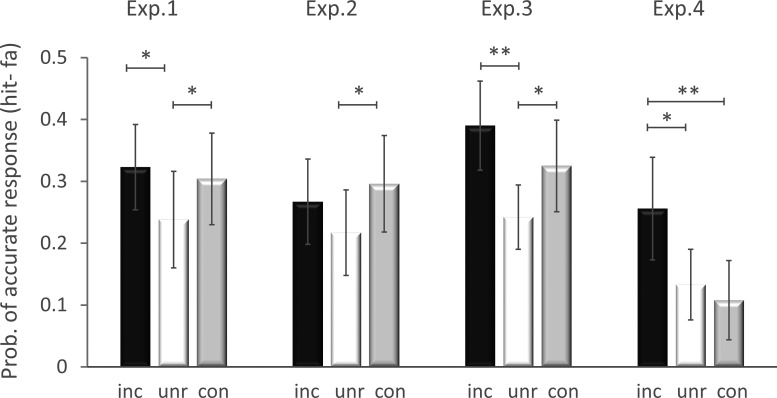
Event memory: last critical trial. Mean (and error bars showing 95% confidence interval) of event memory across Experiments 1–4 for the last Study trials for which memory was tested. High confidence responses are shown in black for incongruent (inc) condition, in white for unrelated (unr) condition, and in gray for congruent (con) condition (for raw data see, https://osf.io/ng3w9/). Note that the last trial tested in the incongruent condition was the third rather than fourth trial in Experiment 2. A U-shaped function was predicted for Experiments 1–3, but not Experiment 4, where no congruency advantage was predicted (see text). Memory accuracy is defined by the proportion of hits minus false alarms, for high confidence responses. * *p* < .05. ** *p* < .01.

As predicted by SLIMM, memory for the final study trial was superior in the incongruent than unrelated condition (inc_4>unr_4: *t*(23) = 2.33, *p* = .02, MD = .09, CI [.02, .15], *d* = 0.48) and in the congruent than unrelated condition (con_4>unr_4: *t*(23) = 2.07, *p* = .03, MD = .07, CI [.01, .12], *d* = 0.42), confirming the predicted U-shaped function of congruency (Figure 3, leftmost plot).[Fig-anchor fig3]

#### Event memory: First trial

**Figure 4 fig4:**
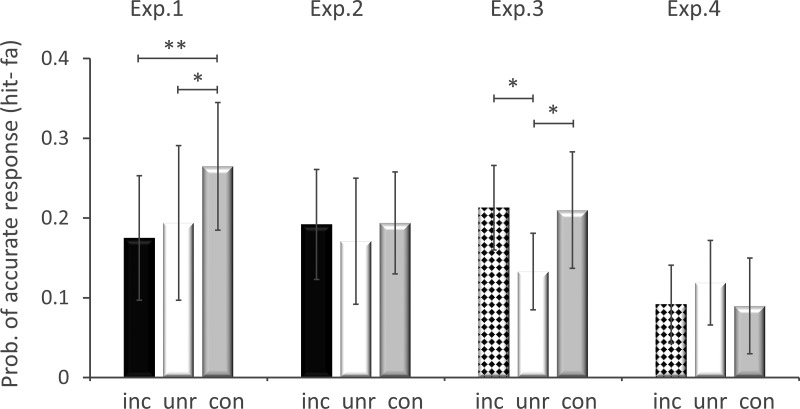
Event memory: first trial tested. Mean (and error bars showing 95% confidence interval) of event memory across Experiments 1–4 for the first trial for which memory was tested. High confidence responses are shown in black for incongruent (inc) condition, in white for unrelated (unr) condition and in gray for congruent (con) condition. Note that the first critical trial tested was the third rather than first trial in the incongruent condition of Experiments 3–4, as distinguished by the checker pattern (see text). Memory accuracy is defined by the proportion of hits minus false alarms, for high confidence responses. * *p* < .05. ** *p* < .01.

Memory for the first study trial was better in the congruent relative to the unrelated condition (con_1>unr_1: *t*(23) = 2.01, *p* = .03, MD = .07, CI [.01, .13], *d* = 0.41) and relative to the incongruent condition (con_1>inc_1: *t*(23) = 2.93, *p* = .004, MD = .09, CI [.04, .14], *d* = 0.60; see Figure 4). Because the conditions were effectively equivalent for the first trial, better memory for first congruent trials suggests that the benefits of schema congruency on memory can also arise after encoding.[Fig-anchor fig4]

### Discussion

Event memory for final trials was a U-shaped function of congruency, with superior memory for incongruent and congruent trials relative to unrelated trials. This supports the first prediction of the SLIMM framework. A second prediction of SLIMM was that the two ends of this U-shape are supported by different mechanisms, with the incongruency advantage arising at encoding, but the congruency advantage also potentially arising postencoding. This prediction was also supported by the data, which showed that event memory for the first trial (before a schema was established or the conditions even differed) did not differ significantly between the incongruent and unrelated conditions, consistent with a schema being necessary to violate before the incongruency advantage emerges, but was higher in the congruent condition than unrelated condition, consistent with a congruency advantage emerging through additional postencoding processes. Such postencoding processes could include consolidation, reactivation or retrieval-related processes; possibilities to which we return in the general discussion.

According to SLIMM, congruent events are more quickly consolidated into memory because they are more likely to be reactivated (or replayed) when a schema is reactivated (because they are consistent with that schema). Thus in the present paradigm, it is likely that the first trial is more often retrieved during the subsequent three study trials in the congruent condition than in the other conditions, further improving its encoding. It is also possible that reactivating the schema in the final test phase improves retrieval of all congruent trials, though it should be noted that knowledge of the schema at test (e.g., remembering that umbrellas had twice the value of shoes) did not on its own enable participants to distinguish between old and new trials in the test of event memory (e.g., whether a specific trial with two umbrellas and one shoe ever occurred at study).

However, an alternative interpretation for the U-shaped function of memory for final trials is that performance in the unrelated condition was impaired relative to the other conditions. For example, participants might become frustrated with the frequent schema reversals in the unrelated condition, causing them to “give up” trying to learn the rule for the object-pair, impairing performance on the final trials in this condition. Alternatively, negative feedback (particularly when surprising) might disrupt memory for preceding trials, and this disruption, which would be most frequent in the unrelated condition, might explain the worse performance on the first trial in the unrelated condition than congruent condition.

We address these two possibilities in the next experiments, reducing the probability that participants give up by having fewer reversals in the unrelated condition (Experiments 2–4), by controlling and measuring the potential influence of distraction (Experiment 3–4), and most importantly, showing above-chance prediction for final study trials in the unrelated condition (Experiments 2–4).

## Experiment 2

To test whether participants give up in the unrelated condition, Experiment 2 introduced two changes: (a) value assignment in the unrelated condition no longer switched between the third and fourth trial, and (b) memory was assessed for the third trial instead of fourth trial (Figure 2b). This not only reduced the total number of schema changes in the unrelated condition, but also meant that we could measure whether participants had given up: If participants were still trying to learn a schema in the unrelated condition, their prediction accuracy for the fourth trial should now be above chance (unlike in Experiment 1). However, to assess memory for unrelated trials in the absence of a consistent schema, like in Experiment 1, we tested memory for the third instead of fourth study trial across all conditions.

### Method

#### Participants

Twenty-four (15 females, mean age 22 years, SD = 4.65 years) volunteers were tested (see Experiment 1 for additional details).

#### Procedure and materials

The procedure was identical to Experiment 1, with the exception of testing event memory for the third rather than fourth study trial, while still presenting a fourth trial in which the same schema was used as in the third trial (Figure 2b). Note that this meant that the total proportion of schema reversals across trials in Experiment 2 (33%) was lower than in Experiment 1 (44%), which should also increase the overall incentive to attend to the schema in all conditions. It also meant that after study, there had been three schema-consistent trials and one schema-inconsistent trial in the unrelated condition, so that schema memory should be above chance at test (unlike Experiment 1, where there were two schema-consistent and two schema-inconsistent trials).

### Results

#### Schema learning and memory

Accuracy during study (see Table 1) significantly increased to above chance on the third trial of the congruent condition (con_3 > .5: *t*(23) = 18.93, *p* < .001, MD = .32, CI [.29, .34], *d* = 3.87). The incongruent condition showed above chance accuracy for the second trial (inc_2 > .5: *t*(23) = 12.17, *p* < .001, MD = .23, CI [.20, .26], *d* = 2.49), which switched to below chance when the schema reversed in the third trial (inc_3 < .5: *t*(23) = −16.27, *p* < .001, MD = −.35, CI [−.39, −.31], *d* = 3.32). The third trial in the unrelated condition was also below chance (unr_3 < .5: *t*(23) = −9.50, *p* < .001, MD = −.30, CI [−.35, −.24], *d* = −1.94). More importantly, accuracy for the fourth unrelated trial, when the schema did not change, was significantly above chance (unr_4 > .5: *t*(23) = 4.96, *p* < .001, MD = .17, CI [.11, .23], *d* = 1.01), indicating that participants were still engaged in trying to learn the schema.

This schema knowledge was maintained at test (last row of Table 1), with significantly higher accuracy for congruent than incongruent new trials, *t*(23) = 5.31, *p* < .001, MD = .16, CI [.11, .22], *d* = 1.09, though not for incongruent than unrelated new trials, *t*(23) = 0.26, *p* = .80, two-tailed, MD = .01, CI [−.04, .06], *d* = 0.05. However, performance for incongruent, *t*(23) = 2.75, *p* = .01, MD = .058, CI [.02, .10], *d* = 0.56, as well as unrelated trials, *t*(23) = 3.30, *p* = .002, MD = .05, CI [.03, .08], *d* = 0.67 was slightly, but significantly, above chance.

#### Event memory: Third trial

Event memory for the third trial (see Figure 3) was significantly better for congruent than unrelated conditions (con_3>unr_3: *t*(23) = 1.79, *p* = .043, MD = .08, CI [.004, .16], *d* = 0.37) and was numerically, but not quite significantly, better for incongruent than unrelated third trials (inc_3>unr_3: *t*(23) = 1.62, *p* = .06, MD = .05, CI [−.003, .10], *d* = 0.33).

#### Event memory: First trial

Analysis of first trials revealed no significant differences between conditions, *t*(23) < 0.60, *p* > .55, two-tailed (Figure 4).

### Discussion

We observed the same general U-shaped function of event memory against congruency for third trials, even when prediction accuracy of the fourth study trial in the unrelated condition was above chance. The latter argues against the alternative possibility considered in Experiment 1 that the U-shaped function reflects decreased memory performance for the unrelated condition because participants ‘give up’ learning the schema.

Although memory for incongruent trials was numerically greater than for unrelated trials, this did not reach significance (unlike in Experiment 1). This is most likely because schemas had not been learned as strongly by the third trial as they had by the fourth trial in Experiment 1. This likely reduced the prediction error, weakening the incongruency advantage. We addressed this in Experiment 3.

In contrast to Experiment 1, there was now a reliable schema in the unrelated condition (obeyed by 3 of the 4 trials). This might explain why the congruency advantage for the first trial was no longer significant. We again addressed this in Experiment 3, which had four aims: (a) to replicate the significance of the U-shaped function by testing the fourth instead of third trial, which should boost prediction strength similar to Experiment 1; (b) to eliminate schema consistency for the first unrelated trial in order to reproduce the congruency advantage for first trials; (c) to maintain a schema from third to fourth trial in the unrelated condition, similar to Experiment 2, to reconfirm task engagement with above chance prediction accuracy at study; and (d) to address the possibility raised in Experiment 1 that frequent negative feedback might be distracting and impair memory for preceding trials in the unrelated condition.

## Experiment 3

Experiment 3 reverted to testing event memory for the fourth trials, thereby increasing schema strength at the point of encoding the critical trials relative to Experiment 2. This should replicate the significant incongruency advantage of Experiment 1 that was only a trend in Experiment 2. The second important change in Experiment 3 was that the schema only reversed once in the unrelated condition, between the first and second trials (Figure 2c). This reduced the total number of reversals compared to Experiments 1 and 2 (and made it the same number as in the incongruent condition), further discouraging participants from giving up. Indeed, the motivation to learn the schema in the unrelated condition could again be measured by prediction accuracy on the fourth study trial.

The single reversal after the first unrelated trial also meant that this trial was now incongruent with the (partial) schema that could be established across Trials 2–4, which should reestablish the advantage for the first trial in the congruent condition seen in Experiment 1. However, the possibility remains that memory for the first unrelated trial is impaired due to the distracting effect of the negative feedback that is likely to follow on the subsequent trial. To test this, we measured memory for the third trial, rather than first trial, in the incongruent condition. The third incongruent trial is also likely to be followed by negative feedback on the subsequent trial, so should show similar memory impairments if such negative feedback plays a key role.

### Method

#### Participants

Twenty-four (14 females, mean age 26 years, sd = 5.23 years) volunteers were tested (for further details see Experiment 1).

#### Materials and procedure

Material and procedure were the same as described in Experiment 1, except that (a) the second rule change in the unrelated condition was omitted, so that Trials 2–4 followed a consistent rule which, in contrast to Experiment 2, rendered the first unrelated trial schema inconsistent, and (b) event memory for the incongruent condition was tested for the third instead of first trial (see Figure 2), to test hypotheses about the distracting effects of negative feedback.

### Results

#### Schema learning and memory

Accuracy at study (see Table 1) reached above chance for the final trial in the congruent condition (con_4 > .5: *t*(23) = 16.21, *p* < .001, MD = .36, CI [.32, .39], *d* = 3.31), but decreased to below chance for the final trial in the incongruent condition (inc_4 < .5: *t*(23) = −23.20, *p* < .001, MD = −.39, CI [−.41, −.36], *d* = −4.74), as expected. However, the unrelated condition now also showed above chance performance for the final trial (unr_4 > .5: *t*(23) = 6.71, *p* < .001, MD = .20, CI [.15, .25], *d* = 1.37). Thus, unlike Experiment 1, participants still acquired a coherent schema across the final three trials in the unrelated conditions, despite the fact that this schema differed from that on the first trial. Nonetheless, accuracy on the final trial in the unrelated condition was still significantly less than that in the congruent condition (unr_4 vs. con_4), *t*(23) = −6.28, *p* < .001, MD = −.16, CI [−.20, −.11], *d* = −1.28.

This schema knowledge was maintained at test (last row of Table 1), with significantly higher accuracy for congruent than unrelated new trials, *t*(23) = 4.48, *p* < .001, MD = .11, CI [.07, .15], *d* = 0.91, though any difference between unrelated and incongruent trials was not significant, *t*(23) = 1.77, *p* = .090, two-tailed, MD = .08, CI [−.01, .17], *d* = 0.40. Performance was significantly above chance for both incongruent, *t*(23) = 4.49, *p* < .001, MD = .15, CI [.10, .21], *d* = 0.92, and unrelated, *t*(23) = 5.90, *p* < .001, MD = .23, CI [.16, .30], *d* = 1.20, new trials.

#### Event memory: Last critical trial (3rd trial)

Memory for the fourth study trial was better in the incongruent than unrelated condition (inc_4>unr_4: *t*(23) = 4.32, *p* < .001, MD = .15, CI [.09, .21], *d* = 0.88) and in the congruent than unrelated condition (con_4>unr_4: *t*(23) = 2.31, *p* = .015, MD = .08, CI [.02, .15], *d* = 0.47), replicating again the predicted U-shaped function of congruency (see Figure 3).

#### Event memory: First trial

Memory for the first study trial was superior in the congruent than unrelated condition (con_1>unr_1: *t*(23) = 2.26, *p* = .017, MD = .08, CI [.02, .14], *d* = 0.46), replicating Experiment 1 (see Figure 4). Memory for the third incongruent trial was also significantly greater than the first unrelated trial (inc_3>unr_1: *t*(23) = 2.32, *p* = .015, MD = .08, CI [.02, .14], *d* = 0.47). If negative feedback from a schema reversal impedes memory for trials directly preceding the reversal, this would be most prominent for strong violations, that is, for incongruent over unrelated trials. Our data show no evidence of such an increased impediment, suggesting negative feedback is not a sufficient explanation for the low memory performance for first trials that precede a schema reversal in the unrelated condition.

### Discussion

Experiment 3 replicated the significant U-shaped function of event memory against congruency for final trials. The advantage for the incongruent versus unrelated condition (left side of U-shape) replicated Experiment 1, and was more reliable than in Experiment 2, as we predicted based on the stronger schema at the time of encoding (by testing fourth rather than third trials). Indeed, this advantage for incongruent relative to unrelated trials occurred even though the total number of schema-congruent trials was the same (three out of four study trials). Prediction accuracy for the fourth unrelated trial was again above chance, like in Experiment 2, suggesting that participants were still motivated to learn a schema and therefore paid attention to the objects in the unrelated condition.

The advantage for first trials in the congruent condition, relative to the unrelated condition, was now significant again (like Experiment 1), most likely because the first trial in the unrelated condition was inconsistent with the dominant schema in that condition (unlike Experiment 2). This is again consistent with schemas influencing memory even after encoding. Finally, memory for the first unrelated trial was worse than for the third incongruent trial, despite both trials being followed by a schema-reversal, suggesting error feedback on the subsequent trial is unlikely to cause distractions that can account for the poor performance on the first trial in the unrelated condition.

Experiments 1–3 suggest that the U-shaped function of event memory against congruency is robust, with no obvious confounding explanations. Moreover, different processes seem to underlie the two ends of this function, because only the congruency advantage remained when testing memory for initial trials, before a schema has been learned (i.e., the left-hand side of the U-shape can be selectively removed). The final experiment sought further evidence for functional dissociation between these two extrema, by making the number of objects an irrelevant detail of the study task. As explained in the introduction, this third prediction of the SLIMM model is based on the idea that prediction errors (as in the incongruent condition) trigger the MTL system to encode all aspects of a surprising event, including those that are irrelevant to the current schema, whereas events congruent with a schema (in the congruent condition) are only encoded in terms of details relevant to the schema. Therefore, if the number of objects is no longer relevant to the schema, memory for this detail should still be better for final trials in the incongruent than unrelated condition, but not differ between the unrelated and congruent condition—that is, the right-hand side of the U-shape should be removed.

## Experiment 4

Experiment 4 had an identical design to Experiment 3 (Figure 2D), except that the study task was to predict which of the two object types had a higher value, regardless of the number of exemplars. According to SLIMM, prediction errors should trigger a complete encoding of the entire event, including incidental information such as the number of exemplars, which should occur for incongruent but not congruent events. We therefore expected an incongruency advantage in the absence of a congruency advantage, that is, inc_4 > unr_4 = con_4, for event memory of final trials.

### Method

#### Participants

Twenty-four (13 females, mean age 24, *SD* = 4.69) volunteers were tested (see Experiment 1 for details).

#### Materials and procedure

Materials and procedure were identical to Experiment 3, except that participants were required to decide whether the objects at the top or bottom had a higher value, irrespective of how many exemplars of those objects were displayed. Participants were told one object in each pair had twice the value of the other (but not which) and that only the assigned value is relevant for performing the task accurately, regardless of the number of exemplars. For instance, for a display of one umbrella on top and two shoes on the bottom, with umbrella assigned the value of 2 and shoe assigned the value of 1, the top display should be selected (top > bottom). Participants therefore only made one of two responses (the top and bottom displays could never have equal value).

### Results

#### Schema learning and memory

Accuracy at study (see Table 1) was above chance for the final trial in the congruent condition (con_4 > .5: *t*(23) = 6.32, *p* < .001, MD = .21, CI [.15, .27], *d* = 1.29), but decreased to below chance for the final trial in the incongruent condition (inc_4 < .5: *t*(23) = −8.32, *p* < .001, MD = −.29, CI [−.23, −.35], *d* = −1.70), as expected. The final trial in the unrelated condition was also above chance (unr_4 > .5: *t*(23) = 2.87, *p* = .004, MD = .06, CI [.02, .10], *d* = 0.59).

The only surprising result was that performance on the second trial in the unrelated condition, following the schema reversal, was not less than chance (.50), as it was in Experiment 3. This could reflect procedural changes between the two experiments, or simply random error. Nonetheless, we do not think this affects the main results, given that the remaining patterns of prediction accuracy, particularly for the critical first and final trials, were as expected.

This schema knowledge was maintained at test (bottom row of Table 1), with significantly higher accuracy for congruent than unrelated new trials, *t*(23) = 4.59, *p* < .001, MD = .18, CI [.11, .25], *d* = 0.94, though any difference between unrelated and incongruent trials failed to reach significance, *t*(23) = 0.35, *p* = .73, two-tailed, MD = .02, CI [−.09, .13], *d* = 0.07. Nonetheless, performance for incongruent, *t*(23) = 4.54, *p* < .001, MD = .15, CI [.09, .20], *d* = 0.93, and unrelated new trials, *t*(23) = 4.21, *p* < .001, MD = .16, CI [.10, .23], *d* = 0.86, was significantly above chance (0.5).

#### Event memory: Last critical trial (4th trial)

Memory for the fourth study trial was better for the incongruent relative to unrelated condition (inc_4>unr_4: *t*(23) = 2.54, *p* = .009, MD = .12, CI [.04, .21], *d* = 0.52) and incongruent relative to congruent condition (inc_4>con_4: *t*(23) = 4.16, *p* < .001, MD = .15, CI [.09, .21], *d* = 0.85), but did not differ significantly between the congruent and unrelated conditions (con_4>unr_4: *t*(23) = −0.77, *p* = .45, two-tailed, MD = −.03, CI [−.09, .04], *d* = −0.16), flattening the right side of the U-shaped function, as predicted (see Figure 3).

#### Event memory: First trial

Memory for the first study trial also failed to show any congruency advantage relative to the unrelated condition (con_1>unr_1: *t*(23) = −0.89, *p* = .19, MD = −.03, CI [−.09, .03], *d* = −0.18), again consistent with event details being irrelevant to the schema (see Figure 4). There was no evidence of greater memory for the third incongruent trial than first unrelated trial (inc_3 > unr_1: *t*(23) = −0.85, *p* = .40, two-tailed, MD = −0.03, CI [−0.09, 0.04], *d* = −0.22), unlike in Experiment 3. This is also consistent with any schema that had been established by the third trial of the incongruent condition no longer being helpful for remembering irrelevant event information as it was in Experiment 3.

### Discussion

Consistent with the third prediction of SLIMM outlined in the introduction, when the number of exemplars was irrelevant to the schema, there was no longer any advantage in event memory for congruent relative to unrelated trials, for first or final trials. Nonetheless, the advantage for incongruent relative to unrelated final trials remained significant (despite schema knowledge at test not differing between these conditions). This supports the claim that different mechanisms underlie the two ends of the congruency dimension.

## General Discussion

We have demonstrated, for the first time within the same paradigm, better memory for the details of events that are either congruent or incongruent with a schema, relative to unrelated events. This U-shaped pattern of event memory as a function of congruency was predicted by the SLIMM framework (van Kesteren et al., 2012), but has not been reported before. We think there are at least two reasons for this. The first reason is theoretical: studies have tended to focus on either schema theory (and the advantage for congruent information) or distinctiveness theory (and the advantage for incongruent information), that is, only ever considered one side of the congruency dimension. The second reason is methodological: most studies have only considered two conditions, for example, memory for congruent versus incongruent information (Bein et al., 2015; Brod et al., 2015), and therefore lacked a third condition, for example, with weak (in)congruency, that is necessary to observe a U-shape. There are other studies that have explored a range of (subjectively defined) congruency levels (e.g., van Kesteren et al., 2013; Lew & Howe, 2017), and found an advantage of congruency. However, the memory tests in these studies allowed prior knowledge (schemas) to aid performance at retrieval. For example, when memory for the event “her handbag contained a wallet” is cued by “handbag,” participants can use prior knowledge to generate objects associated with handbags and then recognize which object seems familiar (Watkins & Gardiner, 1979). By testing memory for information (number of objects) that could not be generated by knowing the schema, we were able to unmask the incongruency advantage (see also Greve et al., 2017, for other ways to avoid use of schema at retrieval).

Not only did our study reveal a U-shaped function of event memory against schema congruency for the first time, but it further supported the SLIMM predictions that qualitatively different mechanisms underlie the two ends of the congruency dimension. We dissociated these two ends of this U-shape in two separate ways: in terms of (a) underlying process, that is, whether the memory advantage arises during or after encoding; and (b) nature of the memory, that is, whether incidental details are also remembered better. In terms of encoding processes, Experiment 1 and 3 showed an advantage for the first trial in the congruent condition relative to other conditions, even though all conditions were indistinguishable at this stage. This suggests that the effect of schema congruency can benefit postencoding processes too. By comparison, prediction errors require schemas to be established, before they can enhance encoding in the incongruent condition. Moreover, the stronger the schema, the bigger the prediction error, which can explain why Experiment 2 showed a weaker incongruency advantage than other experiments after presenting only two rather than three congruent trials.

In terms of the nature of the memory, Experiment 4 abolished the congruency advantage, without affecting the incongruency advantage, by rendering event details irrelevant to the schema. This is consistent with SLIMM’s claim that prediction errors prompt memory processes that engage the medial temporal lobes (MTL) to encode all details of the current event, even if irrelevant to the current schema. This complete encoding is likely to have adaptive value because the particular detail(s) causing the prediction error might become apparent in future, potentially requiring an update or change of schema. Thus, the U-shaped function of congruency does not reflect a single quantitative factor (e.g., lack of attention to inconsistent schema, as tested in Experiment 2–3), but rather qualitatively different memory processes operating at each extreme.

As noted in the introduction, there are several related theories concerning the memory advantage for unexpected events, such as distinctiveness (Hunt & McDaniel, 1993; Hunt & Worthen, 2006; Murdock, 1960). This theory allows for distinct items to benefit at retrieval (e.g., for isolates at the start of lists). Although Schmidt and Schmidt (2017) demonstrated that memory benefits for unexpected items (isolates) arise at encoding as well as retrieval, the SLIMM framework does not address the latter, that is, how isolates benefit from retrieval processes (Hunt & McDaniel, 1993; Rajaram, 1996; Reder, Donavos, & Erickson, 2002). However, we suspect that such retrieval effects require the content of the unexpected event to be distinct from that of other events. In the present paradigm, the content tested by our event memory test (the number of objects) is no more distinct for unexpected than expected trials, at least in the sense that the incongruent trials are not perceptually or conceptually distinct (what Schmidt, 1991, called *primary distinctiveness*). What renders a trial unexpected is simply whether that content violates the currently active schema (more akin to Schmidt’s “secondary distinctiveness”). Moreover, because trials were intermixed across conditions, the overall rate of negative feedback was relatively high, such that feedback itself is an unlikely source of distinctiveness. Incongruent events were not temporally distinct either. Thus, unexpected trials do not necessarily “stand out” as different at the time of test, which is why we think they are better explained by encoding-related prediction error than the more generic notion of distinctiveness.

A similar issue arises in relation to theories of “novelty” in memory (e.g., Tulving & Kroll, 1995). As argued in Henson and Gagnepain (2010), prediction error is more than just novelty, given that a novel item in a novel context might constitute maximum novelty, but have no prediction error (because the novel context provides no schema for predictions). Moreover, the event information tested in the present paradigm is not inherently novel; again, what is “novel” is whether the type of feedback is expected or unexpected. Thus we doubt that the present results can be accommodated by the “novelty-encoding hypothesis” (Greene, 1999; Kinsbourne & George, 1974; Kormi-Nouri, Nilsson, & Ohta, 2005; Tulving & Kroll, 1995). Indeed, subsequent work has cast doubt on this effect being truly encoding-related, pointing instead toward higher retrieval costs for familiar than novel items, that is, increased source confusion and false alarm rates (Dobbins et al., 1998). After controlling for such potential confounds, studies have shown better memory for familiar than novel items (Poppenk, Köhler, & Moscovitch, 2010; Poppenk, McIntosh, Craik, & Moscovitch, 2010).

Given that the content of our incongruent trials was neither novel nor distinct, we propose that it was the prediction errors elicited by unexpected type of feedback that drove better encoding, consistent with studies examining the role of such corrective feedback in learning (Butterfield & Metcalfe, 2001, 2006; Fazio & Marsh, 2009; Metcalfe, 2017). Interestingly, errors made with high confidence are more likely to be remembered accurately after corrective feedback than errors made with low confidence, a finding termed the *hypercorrection effect* (Butterfield & Metcalfe, 2001). High-confidence errors are thought to attract higher levels of attention due to the discrepancy between subjective assessment and performance. Studies testing this hypothesis using divided attention paradigms have shown a decline in secondary task performance when feedback for high confidence errors are processed, confirming increased attentional resources and suggesting that more sustained processing of the corrective information is the cause of this effect (Butterfield & Metcalfe, 2001, 2006; Fazio & Marsh, 2009). Nevertheless, although attention may mediate the improved learning, the underlying cause must be an initial prediction error (Greve et al., 2017; Henson & Gagnepain, 2010). Interestingly, the confidence of a prediction tends to be correlated with how much a participant knows about the target domain (Butterfield & Metcalfe, 2001) and a study by Sitzman, Rhodes, and Tauber (2014) demonstrated that prior domain knowledge, and not response confidence per se, increases the likelihood of incorporating new information into memory, which is consistent with the present findings.

Regarding the other end of the congruency continuum—the congruency effect—this is consistent with many previous findings (e.g., Atienza et al., 2011; Alba & Hasher, 1983; Bein et al., 2015; Cycowicz et al., 2008; Craik & Tulving, 1975), including reports of better memory for words that have preexisting semantic, associative or thematic relationships (Craik & Tulving, 1975; Kriukova, Bridger, & Mecklinger, 2013; Roediger & McDermott, 1995). For example, recognition memory studies show superior memory for related than unrelated word pairs (Greve, van Rossum, & Donaldson, 2007; Rhodes & Donaldson, 2007) and amnesic patients exhibit impaired associative memory for randomly paired words, but preserved recognition of highly related words (Cutting, 1978; Shimamura & Squire, 1984; Winocur & Kinsbourne, 1978). This mnemonic advantage is thought to reflect facilitated acquisition of related items because they activate and strengthen preexisting semantic associations, while unrelated items require the creation of new associations (Wickelgren, 1979). Although this resonates with the mnemonic advantages for schema-congruent items found here, an additional characteristic that distinguishes schemas from preexisting semantic associations is their ability to abstract and therefore generalize to new situations (e.g., for a rule to be applied to new trials in the present paradigm). A recent study investigated whether schemas support the mnemonic advantage that arises when a new relationship between words is made explicit through a definition, in order to encourage their binding as a “unitized” representation. However, the authors found no evidence that this representation generalized to other semantically related words, contrary to what would be expected if a schema had been formed, which suggests that unitization is different from schematization (Tibon, Greve, & Henson, 2018). Future work is needed to distinguish the simple reactivation of prior semantic associations from the more flexible abstraction of a schema and from the less flexible recoding of associations into single units.

It is important to note that our design meant that memory for the schema (rule) could not help retrieve a specific trial at test, in that knowledge of the rule would not, on its own, allow one to guess whether a specific combination of exemplars had been studied (i.e., we controlled for a “generate-and-recognize” strategy), suggesting that the congruency advantage does not arise from retrieval-related processes at test. Rather, the postencoding congruency advantage seems more likely to arise from consolidation or reactivation processes operating between encoding and retrieval. Systems-level consolidation is believed to involve gradual reorganisation over an extended period of time (Frankland & Bontempi, 2005), so is unlikely to occur over the brief study-test delay used here. The congruency advantage is more likely, we think, to occur during the study phase, reflecting the reactivation of previous congruent trials during the encoding of new trials, resulting in additional encoding opportunities for congruent trials (see also van Kesteren et al., 2012).

This mechanism mediating the congruency advantage for first trials may relate to the within-list primacy effects found for categorized lists: In previous studies, lists containing blocks of items from the same category (e.g., flowers, animals, countries, etc.) reveal a memory advantage for the first item within each block, even if those items occurred in the middle of the list as a whole (e.g., Gorfein, Arbak, Phillips, & Squillace, 1976). This effect has been attributed to increased rehearsal of items from the same category, for which the category name becomes an implicit associative response: The later occurrence of an item cues its category name, which reactivates earlier items of the same category, improving their encoding (Underwood & Freund, 1969; Wood & Underwood, 1967). Learning of schema congruent information might operate in similar ways. Our paradigm repeatedly presents the same object pairs within the same conditions. The second encounter of a pair might reactivate an earlier memory of that pair, which strengthens it and makes it more likely to be remembered. Although this benefit should occur across all conditions, trials that share not only the same objects but also the same rule or schema might have an added advantage of enhanced reactivation. Future studies are needed to test this possible explanation more closely.

In general, one could argue that our simple rules, learned during the course of the experiment, do not conform to the concept of schemas used in previous research (see Ghosh & Gilboa, 2014 for review). Schemas are usually conceived as rich and complex abstract structures that summarize knowledge about the real-world, and that are used, for example, in reconstructing autobiographical memories, as suggested by the basic-systems model (Rubin, 2006). Furthermore, the schemas used in many previous studies normally exist pre-experimentally and were acquired and consolidated over many days or years (Anderson, 1981; Gilboa & Marlatte, 2017; Kole & Healy, 2007; Schank & Abelson, 1977; Stangor & McMillan, 1992). However, by distilling the concept to its minimal features of an abstract structure that can influence encoding of new information (the individual trials), we would argue that we were able to achieve more experimental flexibility and control than in previous studies. For example, only by training new schemas during the experiment could we examine their effect on memory for event information presented before they had been established. Our concept of schema was influenced by the neuroscientific perspective offered by SLIMM (van Kesteren et al., 2012), as the set of currently active cortical representations than influence online processing. Even if one would prefer to reserve the term *schema* for more complex, real-world knowledge, we believe our results still reveal important insights on the factors determining event memory. Future work could test whether the findings here generalize to more established, complex structures.

More research is also needed to investigate the brain mechanisms underlying the qualitatively distinct mechanisms proposed here. For instance, the neural underpinnings of the behavioral U-shape function obtained in Experiment 3 could be investigated using functional MRI. According to SLIMM, prediction errors (i.e., fourth trial in the incongruent condition) are expected to increase activity in the MTL in order to store a complete representation of the surprising event, including any incidental information. Highly congruent items (i.e., fourth trial in the congruent condition), on the other hand, should elicit activation in mPFC, to facilitate rapid cortical learning of the new event. Furthermore, for high levels of congruency, the mPFC is believed to suppress MTL activity so that novel perceptual details that are unrelated to the activated schema will not be encoded, a prediction which can be tested by using effective connectivity. The behavioral patterns could also be tested in patients with selective lesions of mPFC or MTL. Patients with mPFC lesions would be predicted to show an attenuated congruency advantage, if their mPFC is unable to use the presence of a schema to enhance cortical learning. The prediction for patients with MTL damage is less clear however. Although they would be predicted to show the complementary pattern of an attenuated incongruency advantage, they may also show an attenuated congruency advantage in the present paradigm, because the paradigm requires learning of new schema (during the study phase), which is also likely to be impaired by MTL damage. Ideally this would be tested by MTL disruption after schema learning, for example by using a variant of the present paradigm in which the schema exist pre-experimentally.

Recently, prediction error models have become increasingly influential in the neuroscience literature and numerous studies have linked the firing of dopamine neurons in the midbrain to the experience of a reward that was not anticipated, that is, a reward prediction error (Bayer & Glimcher, 2005; Eshel et al., 2015; Schultz, Dayan, & Montague, 1997). The phasic firing of dopamine neurons relates to the size and subjective value of the unexpected reward (Eshel, Tian, Bukwich, & Uchida, 2016; Hollerman & Schultz, 1998; Roesch, Calu, & Schoenbaum, 2007); it shows an increased level of activity when a reward is greater than predicted (positive prediction error), remains at baseline when a reward is fully predicted and reveals depressed activity when a reward is less than predicted (negative prediction error). Recent studies have identified a network of brain regions involved in new learning on the basis of this error signal, namely a dopamine-dependent loop which includes the ventral striatum, the substantia nigra/ventral tegmental area (SN/VTA), and the hippocampus (SN/VTA-HC loop; Lisman & Grace, 2005; Lisman, Grace, & Duzel, 2011; Shohamy & Adcock, 2010). Importantly, the evidence for this network is based mainly on reinforcement learning paradigms, that is, when learning is motivated by external rewards, whereas our paradigm elicits prediction errors in the absence of external rewards. Although some studies suggest that internally driven learning can in itself be rewarding (Ripolles et al., 2014), and that intrinsic motivational states (e.g., curiosity) can support memory formation by engaging the SN/VTA-HC loop (Gruber, Gelman, & Ranganath, 2014), it remains to be tested whether the prediction errors elicited in paradigms like ours engage similar dopaminergic mechanisms. Future studies could examine how external reward might enhance memory in our paradigm. If dopamine is involved, we would predict suppressed dopamine for incongruent trials, elicited by the negative prediction error which weakens future expectation of the incorrectly predicted outcome, but no change in dopamine for schema congruent trials (i.e., critical fourth trial), for which a correct response/reward is fully predicted. Thus it is not obvious how a dopaminergic network could fully explain the superior memory we observe at both extremes of the congruency spectrum.

Taken together, our findings bring together two strands of psychological research: one concerning schema and congruency and another concerning distinctiveness, novelty and prediction error. Furthermore, they relate these strands to recent neuroscientific research about the brain systems supporting memory. More specifically, we tested and confirmed the predictions for a U-shaped function with dissociable tails that was predicted in advance by van Kesteren et al. (2012) in terms of the neuroscientific SLIMM framework. This framework postulates distinct brain systems to handle the opposing demands of, on the one hand, benefitting from reoccurring regularities and schema to enable efficient encoding of our environment, and on the other hand, of accommodating surprising information that does not match prior expectations, which is essential for flexible adaptation to an ever-changing environment.

## Supplementary Material

10.1037/xge0000498.supp

## Figures and Tables

**Table 1 tbl1:** Mean (With 95% Confidence Interval in Brackets) of Proportion of Correct Responses Across All Four Trials (Rep) at Study (Rows 1–4) and of Schema Memory for New Trials at Test as a Function of Each Condition—Incongruent (Inc), Unrelated (Unr) and Congruent (Con)—in Each Experiment

Experiment	Inc	Unr	Con
Experiment 1			
Rep			
1	.46 (.05)	.47 (.05)	.42 (.04)
2	.79 (.09)	.18 (.05)	.82 (.07)
3	.79(.07)	.53 (.09)	.84 (.07)
4	.13 (.05)	.37 (.08)	.87 (.07)
Test	.73 (.07)	.52 (.07)	.85 (.08)
Experiment 2			
Rep			
1	.42 (.04)	.35 (.06)	.40 (.05)
2	.73 (.04)	.14 (.05)	.76 (.05)
3	.15 (.04)	.20 (.06)	.82 (.03)
4	.64 (.08)	.67 (.07)	.88 (.04)
Test	.56 (.04)	.55 (.03)	.72 (.05)
Experiment 3			
Rep			
1	.47 (.04)	.40 (.05)	.43 (.05)
2	.73 (.07)	.19 (.04)	.78 (.05)
3	.80 (.05)	.62 (.06)	.80 (.05)
4	.12 (.03)	.70 (.06)	.86 (.04)
Test	.65 (.07)	.73 (.08)	.84 (.06)
Experiment 4			
Rep			
1	.48 (.03)	.53 (.06)	.41 (.06)
2	.76 (.08)	.57 (.07)	.81 (.06)
3	.88 (.04)	.47 (.06)	.80 (.05)
4	.21 (.07)	.56 (.04)	.71 (.07)
Test	.65 (.06)	.66 (.08)	.85 (.05)
*Note*. For raw data, see https://osf.io/ng3w9/.			

**Table 2 tbl2:** Mean (and 95% Confidence Interval in Brackets) Performance at Test for High Confidence Responses (for Data Collapsed Across Confidence, See Online Supplemental Material)

Experiment	Inc	Unr	Con
Experiment 1			
Repetition			
1	.28 (.10)	.28 (.11)	.35 (.10)
2	—	—	—
3	—	—	—
4	.43 (.09)	.32 (.10)	.39 (.09)
New (FA)	.11 (.06)	.08 (.05)	.09 (.05)
Experiment 2			
Repetition			
1	.32 (.07)	.30 (.08)	.33 (.08)
2	—	—	—
3	.39 (.10)	.34 (.09)	.44 (.09)
4	—	—	—
New (FA)	.13 (.05)	.13 (.05)	.14 (.07)
Experiment 3			
Repetition			
1	—	.28 (.08)	.33 (.09)
2	—	—	—
3	.31 (.08)	—	—
4	.48 (.09)	.39 (.08)	.44 (.09)
New (FA)	.09 (.05)	.15 (.06)	.12 (.05)
Experiment 4			
Repetition			
1	—	.33 (.10)	.33 (.10)
2	—	—	—
3	.29 (.09)	—	—
4	.46 (.11)	.34 (.09)	.35 (.10)
New (FA)	.20 (.08)	.21 (.08)	.24 (.10)
*Note*. Performance is shown as proportions of hits to studied items (Repetitions 1–4) and false alarms (FA) to new items, for each condition: incongruent (Inc), unrelated (Unr), and congruent (Con).
