# Supplementary material for: Knowledge Is Power: Prior Knowledge Aids Memory for Both Congruent and Incongruent Events, but in Different Ways
Source: J Exp Psychol Gen. 2018 Nov 5;148(2):325–41. doi: 10.1037/xge0000498 (PMC6390882; doi:10.1037/xge0000498)
Supplement: Supplementary file 1 [file Knowledgesupplrev2407.zip › Knowledgesupplrev2407.docx]

**Supplemental Material**

**Performance collapsed across confidence levels**

|  |  | ***Experiment 1*** |  |  |  | ***Experiment 2*** |  |
| --- | --- | --- | --- | --- | --- | --- | --- |
| ***repetition*** | ***inc*** | ***unr*** | ***con*** |  | ***inc*** | ***unr*** | ***con*** |
| 1 | .65 (.07) | .64 (.06) | .72(.06) |  | .69 (.05) | .67 (.07) | .74 (.06) |
| 2 | - | - | - |  | - | - | - |
| 3 | - | - | - |  | .77 (.05) | .73 (.06) | .77 (.05) |
| 4 | .83 (.04) | .75 (.04) | .79 (.05) |  | - | - | - |
| new (fa) | .38 (.06) | .37 (.06) | .37 (.06) |  | .44 (.07) | .51 (.09) | .45 (.07) |
|  |  |  |  |  |  |  |  |
|  |  |  |  |  |  |  |  |
|  |  | ***Experiment 3*** |  |  |  | ***Experiment 4*** |  |
| ***repetition*** | ***inc*** | ***unr*** | ***con*** |  | ***inc*** | ***unr*** | ***con*** |
| 1 | - | .69 (.07) | .74 (.06) |  | - | .73 (.06) | .71 (.07) |
| 2 | - | - | - |  | - | - | - |
| 3 | .71 (.07) | - | - |  | .66 (.07) | - | - |
| 4 | .83 (.04) | .78 (.05) | .78 (.06) |  | .84 (.05) | .72 (.06) | .74 (.06) |
| new (fa) | .39 (.07) | .47 (.07) | .44 (.07) |  | .52 (.08) | .56 (.07) | .64 (.04) |

**Supplemental Table 1**: Mean (and 95% Confidence Interval in brackets) performance at Test, collapsing across all confidence levels (for raw data see <https://osf.io/ng3w9/>). Performance is shown as proportions of hits to studied items (repetitions 1-4) and false alarms to new items, for each condition: Incongruent (inc), Unrelated (unr) and Congruent (con).


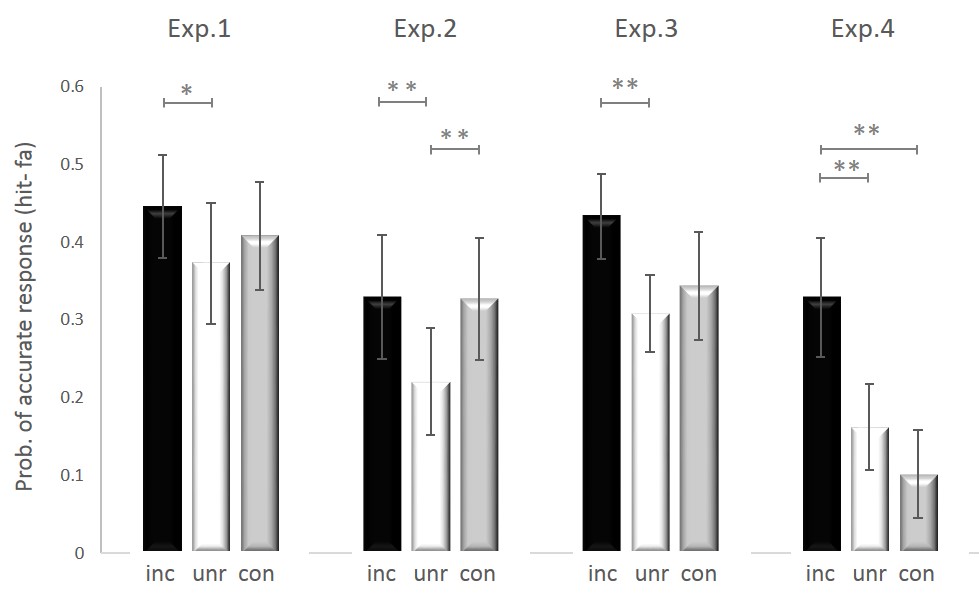


**Supplemental Figure 1: Episodic memory: last critical trial.** Mean (and error bars showing 95% confidence interval) of episodic memory across Experiments 1-4 for the last trials for which memory was tested. Data collapsed across all confidence levels are shown in black for Incongruent (inc) condition, in white for Unrelated (unr) condition, and in grey for Congruent (con) condition (for raw data see <https://osf.io/ng3w9/>). Note that the last trial tested in the Incongruent condition was the third rather than fourth trial in Experiment 2. Memory accuracy is defined by the proportion of hits minus false alarms, for high confidence responses. * = p<.05, ** = p<.01.


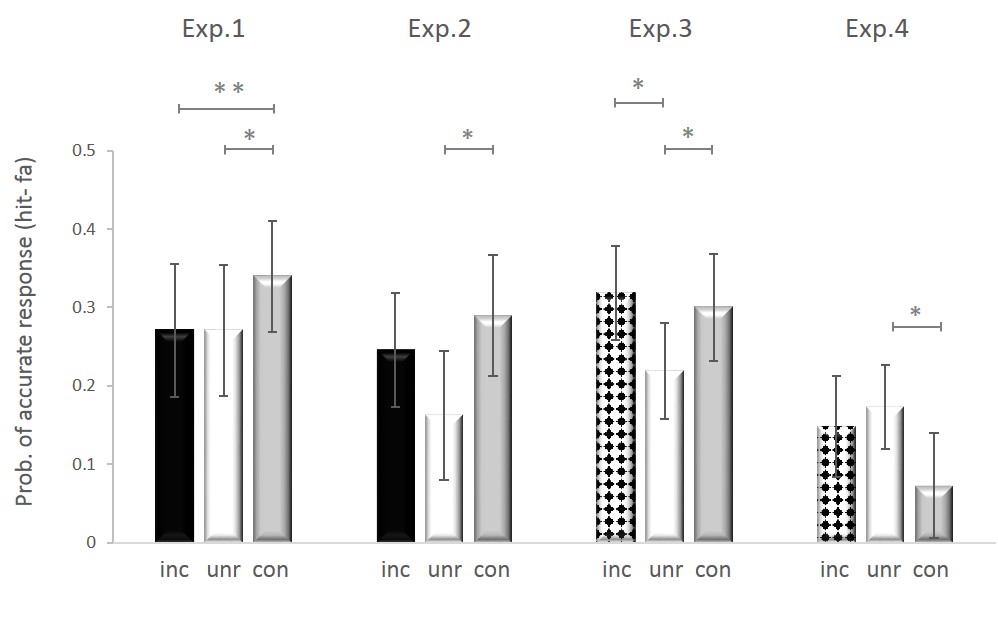


**Supplemental Figure2: Episodic memory: first critical trial.** Mean (and error bars showing 95% confidence interval) of episodic memory across Experiments 1-4 for the first trials for which memory was tested. Data collapsed across all confidence levels are shown in black for Incongruent (inc) condition, in white for Unrelated (unr) condition, and in grey for Congruent (con) condition (for raw data see <https://osf.io/ng3w9/>). Note that the first trial tested was the third rather than first trial in the Incongruent condition of Experiments 3-4, as distinguished by the checker pattern (see text). Memory accuracy is defined by the proportion of hits minus false alarms, for high confidence responses. * = p<.05, ** = p<.01.
